# Editorial: Bench Research Behind Lung Cancer Surgery

**DOI:** 10.3389/fsurg.2022.936922

**Published:** 2022-06-21

**Authors:** Luca Bertolaccini, Lorenzo Spaggiari, Jessica Cusato

**Affiliations:** ^1^ Department of Thoracic Surgery, IEO, European Institute of Oncology IRCCS, Milan, Italy; ^2^ Department of Oncology and Hemato-Oncology, University of Milan, Milan, Italy; ^3^ Department of Medical Sciences, University of Turin, Turin, Italy

**Keywords:** lung cancer, thoracic surgery, bench and pilot test, methodology, basic research

**Editorial on the Research Topic**

Bench Research Behind Lung Cancer Surgery

## Introduction

Lung cancer is the leading cause of cancer death and has remained a major worldwide health problem for decades. Nonetheless, significant progress is being made in preventing and treating lung cancer. With multiple notable achievements, lung cancer therapy has emerged as a role model for personalised cancer medicine. Everything occurs circularly: revolution, change, adaptation to change, acceptance of the new standard, organisation of the new establishment, resistance to further change, revolution, and so forth. This cycle has been recurring every 100 years in surgery, but there has been a noticeable acceleration recently. New scientific developments may enable thoracic surgeons to enhance their current skill set or conduct new treatments outside the traditional scope of thoracic surgery. Despite this, science is advancing faster than ever before, and we are on the verge of another revolution. This is a period in the history of thoracic surgery when genuinely revolutionary change is occurring at an unprecedented rate. While it is true that each generation of physicians significantly outperforms the previous generation in terms of accomplishments, the degree of actual changes is tremendous. The surgeon of the future will need to learn a more excellent range of modern sciences rapidly. Although most people consider the exact sciences unlike more experimental surgery, modern surgery is founded on them.

Fundamental sciences may have a crucial role to play in lung cancer surgery. Medical advances have been made possible by the rapid development of physics and biology, which is underpinned by mathematics: the development of the microscope, the discovery and use of radiation for diagnosis and therapy, and the explosion of clinical laboratory investigations for diagnosis and prognosis. Therefore, every thoracic surgeon should have a working knowledge of the underlying sciences to develop a three-dimensional perspective on surgery as more than a sequence of mechanical actions and as a precision science. Because science, or how things operate, is where technology, or how things work, originates. This Research Topic undertook the audacious task of inquiring into the sciences behind Thoracic Surgery by integrating biologists and medical practitioners and fostering a productive dialogue between all of these disciplines. All of this is in the belief that mutual exchanges can advance knowledge and advancement in Thoracic Surgery.

## From the Bench Research …

Lung cancer is a complex environment composed of cancer cells with changed genomes, a broad array of differentiated cells, and non-cancerous stroma. Organoids of lung cancer are three-dimensional structures generated from patient cancer tissue that can imitate the malignancy’s complicated activity and cellular architecture in vivo. Organoids could be utilised to examine the development of lung cancer, carcinogenesis, and signalling pathways. Additionally, the organoids may be used for drug screening and biomarker validation in lung cancer. A study highlighted how lung cancer organoids had facilitated the translation of basic cancer research into clinical therapy and the present and future breakthroughs in organoid technology that may be applied to lung cancer research (1).

In this manuscript, small ubiquitin-like modifier-specific protease 1 (required for cancer progression and chemoresistance) was demonstrated as a biomarker to optimise the management of surgical non-small cell lung cancer (NSCLC) patients receiving adjuvant chemotherapy [in press].

## … To the Lung Cancer Surgery

The usefulness of targeted therapy in neoadjuvant NSCLC with stage IIIA epidermal growth factor receptor (EGFR) mutations is still debatable. In the neoadjuvant setting, a meta-analysis demonstrates that targeted treatment is superior to chemotherapy in terms of toxicity and tumour response rate in EGFR positive stage IIIA NSCLC (2).

In 1995, the Lung Cancer Study Group published a randomised clinical trial establishing lobectomy as the gold standard for treating early lung cancer. However, some retrospective investigations have demonstrated that pulmonary segmentectomy is as effective as lobectomy. Pulmonary segmentectomy is a surgical technique for select patients with lung cancer since it represents a revolutionary approach to lung cancer treatment and patient survival. Thus, this manuscript aims to summarise the indications for segmentectomy (3).

The optimal time to intervene with small indeterminate pulmonary nodules has long been a point of contention, owing to the low prevalence of malignancy and the difficulty of getting a definitive preoperative diagnosis. This paper compared the survival results of surgical and non-surgical care in NSCLC <8 mm, which could guide prospective decision-making in patients with suspected NSCLC. In patients with <8 mm NSCLC, surgical resection had a better prognosis than non-surgical treatment. However, the factors that should have been incorporated into the proper propensity-matched analysis, such as comorbidity, cardiopulmonary function, and performance status, were not available, and the actual superiority or inferiority should be investigated further in an ongoing randomised trial, particularly when comparing surgery and stereotactic body irradiation (4).

For malignant diseases, sternal excision and anterior chest wall rebuilding procedures are not generally uniform. Reconstruction with titanium mesh enabled rapid parietal stability restoration, improving patients’ respiratory dynamics and recovery (5).

Oligometastatic disease in lung cancer is not a rare condition as previously thought. The authors analysed synchronous isolated cranial metastases patients treated with locally ablative treatments (surgery, radiotherapy, or both). Prognostic factors affecting survival are evaluated retrospectively to identify clinical factors predicting survival to better select patients for surgery. Patients with T1–T2 primary lung tumours, no mediastinal lymph node metastasis, minor anatomical lung resection, neoadjuvant chemotherapy, single cranial metastasis, and surgical cranial metastasectomy were better survival. According to tumour histology, they had adenocarcinoma and not having lymphovascular or visceral pleura invasion correlated with better survival (6).

## Conclusions

Outstanding authors have brought another valuable educational resource with the latest discoveries in this thematic issue of Frontiers in Surgery to keep busy practitioners, scientists, and everyone interested in lung cancer current. In this context, the various manuscripts are not intended to be exhaustive reviews but rather to cover the highlights and provide relevant references for additional reading.

## Author Contributions

Equal contribution status. All authors contributed to the article and approved the submitted version.
